# A case of adenomyoepithelioma with myoepithelial carcinoma of the breast

**DOI:** 10.1002/ccr3.2100

**Published:** 2019-03-30

**Authors:** Ryota Ito, Daisuke Ota, Sumiyo Ando, Masaya Mori, Atsushi Fukuuchi

**Affiliations:** ^1^ Department of Breast and Endocrine Surgery Mitsui Memorial Hospital Tokyo Japan; ^2^ Department of Diagnostic Pathology Mitsui Memorial Hospital Tokyo Japan

**Keywords:** adenomyoepithelioma, breast cancer, myoepithelial carcinoma

## Abstract

Adenomyoepithelioma with myoepithelial carcinoma of the breast is rare and diagnosed with histology and immunohistochemistry. We present a case of malignant transformation over 10 years, with ultrasonographic findings, highlighting the importance of an early excisional biopsy. Conservative surgery and radiation therapy were performed. There was no recurrence for 2 years.

## 1 INTRODUCTION

Normal breast tissue consists of branching ductal networks that are lined by an outer layer of myoepithelial cells and an inner layer of polarized ductal epithelium.^1^ Breast myoepithelial cells are usually located between the basement membrane and breast ductal epithelium.^2^ Based on the World Health Organization classification of tumors of the breast, the myoepithelial lesions are composed of a pure or dominant population of myoepithelial cells, while the epithelial‐myoepithelial lesions are derived from a dual population of epithelial and myoepithelial cells.^3^ Epithelial‐myoepithelial lesions include pleomorphic adenomas, adenomyoepitheliomas (AMEs), AME with carcinoma, and adenoid cystic carcinomas.^3^

Adenomyoepitheliomas is characterized by the bicellular proliferation of glands that consist of an inner epithelial and an outer myoepithelial layer of cells.^4^ It can have a spindle, tubular, or lobulated histological pattern.^5^ One or both of the components of the tumor may exhibit malignant transformation, which is called AME with carcinoma and is characterized by a higher mitotic rate, infiltrative growth pattern, marked cytologic atypia, necrosis, and/or metastasis of cells.^5^

Adenomyoepitheliomas with myoepithelial carcinoma of the breast is rare, and its biological behavior and treatment are not well characterized. This report presents a case of AME with myoepithelial carcinoma of the breast.

## 2 CASE REPORT

**Figure 1:**
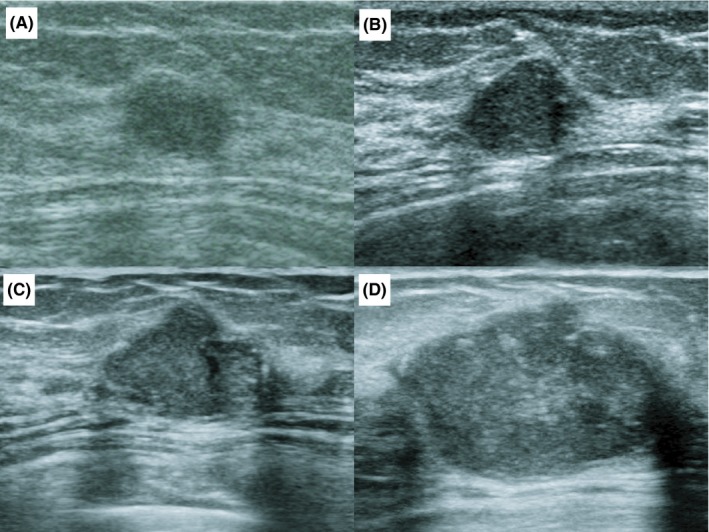
Ultrasonography of a lump in the left breast. Ultrasonography (A) at the initial visit shows a flat and well‐defined hypoechoic lesion measuring 0.8 cm (BI‐RADS category 2), (B) 8 y after the initial visit shows a homogenous hypoechoic lesion measuring 1.5 cm with posterior acoustic shadow, slightly taller than wide ratio and lobulation (BI‐RADS category 5), (C) 9 y after the initial visit shows a more lobulated hypoechoic lesion measuring 1.7 cm (BI‐RADS category 5), and (D) 10 y after the initial visit shows a solid irregular lobulated heterogeneous hypoechoic lesion with a partially unclear border measuring 2.5 cm (BI‐RADS category 5)

A 58‐year‐old postmenopausal woman visited our hospital with a palpable lump in the left breast. A movable lump with a clear border and no tenderness was revealed in the outer quadrant of the left breast. The mammogram revealed only round macrocalcification and no mass lesion (BI‐RADS: Breast Imaging Reporting and Data System category 1; this mammographic image had not been preserved). Ultrasonography revealed the lump to be a flat and well‐defined hypoechoic lesion measuring 0.8 cm in diameter (BI‐RADS category 2; Figure 1A). At the initial visit, following a core needle biopsy (CNB), the lump was diagnosed as a benign epithelial neoplasm. Ultrasonography 2 years later revealed no increase in tumor size. An annual observation during health examination was recommended to monitor the lump. Eight years later, the lump had increased in size, and she visited our hospital again. Ultrasonography revealed a homogenous hypoechoic lesion measuring 1.5 cm with posterior acoustic shadow, slightly taller than wide ratio and lobulation (BI‐RADS category 5; Figure 1B). However, the cytological diagnosis of the CNB did not change. Since malignancy could not be ruled out by the ultrasonographic findings, we recommended the excisional biopsy, but she refused it. In the ninth year, the tumor was 1.7 cm and more lobulated in shape (BI‐RADS category 5; Figure 1C). CNB was performed again, but the diagnosis did not change. She had no symptoms except for the palpable left breast lump. Despite our recommendation, she refused to have an excisional biopsy.

**Figure 2:**
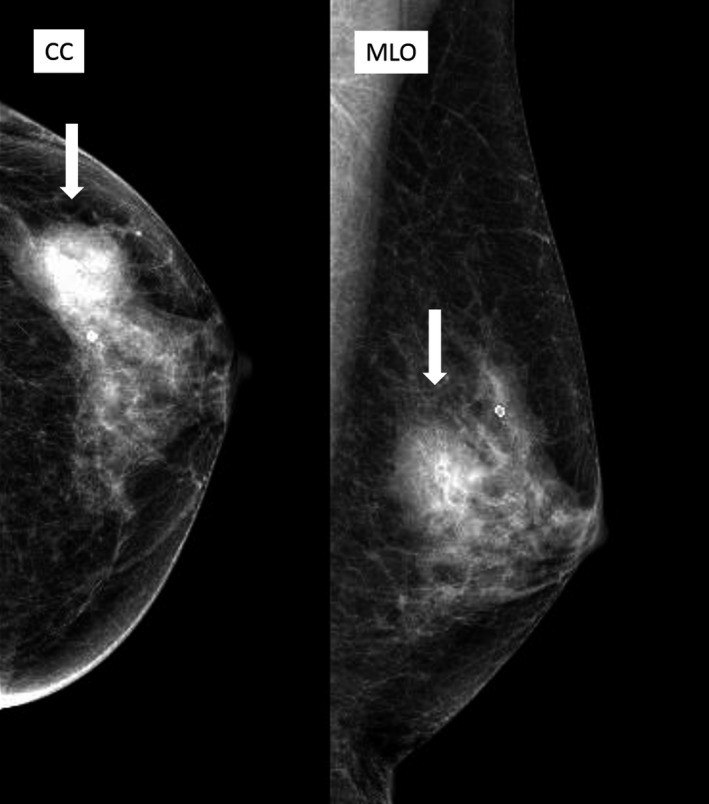
Preoperative left mammography. Shown is a high‐density, well‐demarcated lesion with round macrocalcification and no spicules in the outer, middle area of the left breast (white arrow; BI‐RADS category 3). CC, craniocaudal view; MLO, mediolateral oblique view

At 10 years, a firm mass measuring 2.5 cm in diameter was observed in the middle outer quadrant of the left breast, with no palpable axillary or subclavicular lymph nodes. Ultrasonography showed that the tumor was a solid irregular lobulated heterogeneous hypoechoic lesion, with a well‐defined border, though a part of it was indistinct (BI‐RADS category 5; Figure 1D). While mammography findings delineated a round and circumscribed lesion with round macrocalcification (BI‐RADS category 3; Figure 2), magnetic resonance imaging (MRI) revealed a lobulated heterogeneous high‐intensity mass with a well‐defined border localized in the left breast measuring 2.6 cm. We recommended an excisional biopsy again, to which the patient agreed.

Intra‐operative findings revealed a firm, movable mass with a clear border. There was no gross surgical injury.

**Figure 3:**
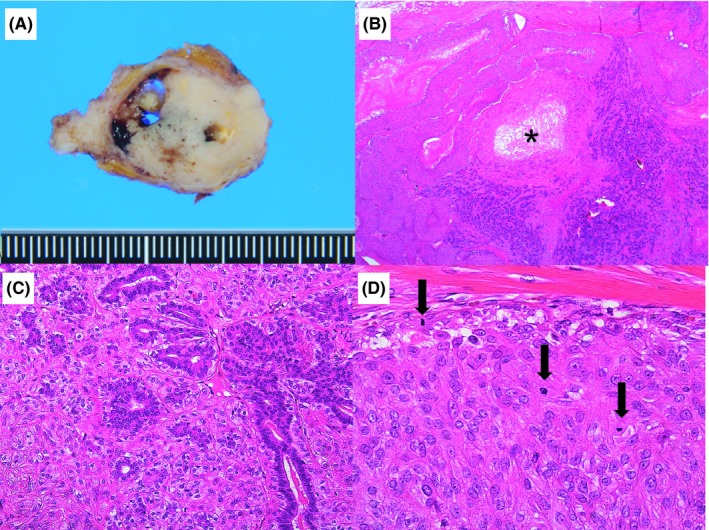
Characteristics of the tumor. A, Macroscopically, the tumor is solid and grayish with focal yellowish parts and cystic degeneration. B, Microscopically, the right side of the figure shows the AME component, while the left side shows the myoepithelial carcinoma component. No separation is seen between the two components. Black asterisk indicates necrosis (×2). C, The AME component shows biphasic proliferation of both ductal epithelium and myoepithelium (×20), and D, the myoepithelial carcinoma component shows monophasic proliferation. The cytoplasm is pale, and the nuclei are pleomorphic and heterogeneous in size. Black arrows indicate mitosis (×40) (B‐D: hematoxylin and eosin stain)

**Figure 4:**
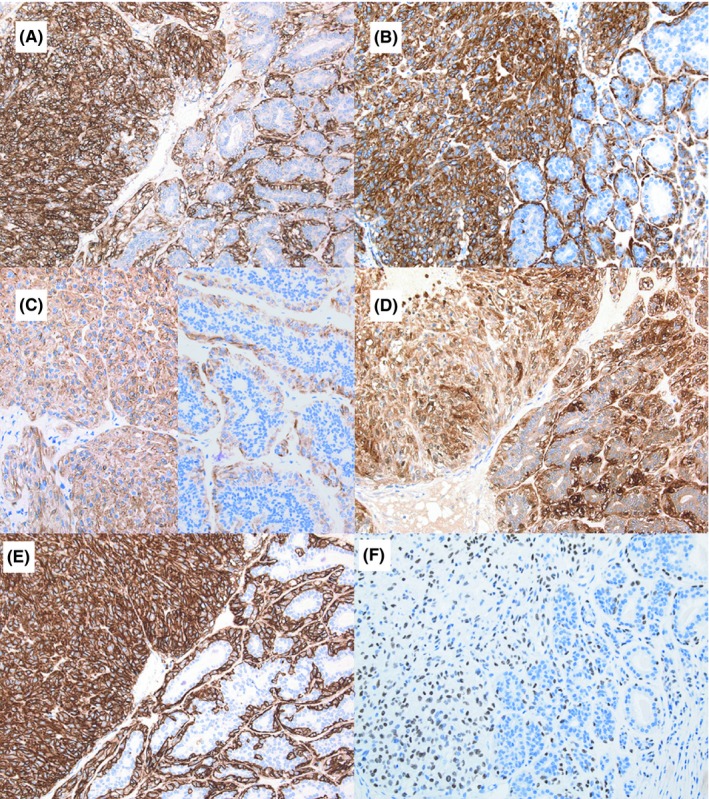
Immunohistochemical analysis. Myoepithelial cells of the AME (right side) and myoepithelial carcinoma (left side) components are positive for (A) HHF35 (×20), (B) α‐SMA (×20), (C) calponin (×20), (D) S‐100 (×20), (E) CD10 (×20), and (F) p63 (×20)

Macroscopically, the tumor measured 2.5 cm in diameter and was a firm, solid mass with areas of cystic and hemorrhagic lesions (Figure 3A). Microscopically, the tumor consisted of two continuous components (Figure 3B). One component showed biphasic proliferation of both ductal and myoepithelial cells (Figure 3C) and was identified as AME. However, the other component showed monophasic proliferation of cells with pale cytoplasm and was similar to the myoepithelium (Figure 3D). This latter component had several mitoses and foci of necrosis (Figure 3D). Immunohistochemistry revealed myoepithelial cells in both the components that were positive for HHF35 (Figure 4A), α‐SMA (Figure 4B), calponin (Figure 4C), S‐100 (Figure 4D), CD10 (Figure 4E), and p63 (Figure 4F), while negative for desmin. Based on these findings, this tumor was identified as AME with myoepithelial carcinoma. Additionally, the myoepithelial carcinoma was found to be negative for estrogen receptor (ER), progesterone receptor (PgR), and human epidermal growth factor receptor (HER) 2. While the Ki‐67 index of the myoepithelial carcinoma was 20%, that of the AME was <5%. Following excision, while the AME component had a positive surgical margin, the myoepithelial carcinoma had a clear margin.

Additional excision of the breast with sentinel lymph node biopsy was performed. Pathological findings showed an absence of cancer cells in the excised specimen, and no metastasis was observed in the sentinel lymph node. After surgery, radiation therapy was delivered with a total dose of 50 Gy in 25 fractions. The tumor has neither recurred locally nor metastasized for up to 2 years after surgery.

## 3 DISCUSSION

Patients with benign AME usually present a solitary, well‐circumscribed, and sometimes palpable nodule.^4^ Patients with myoepithelial carcinoma present the same features, along with tenderness.^1^ In this case, the only clinical presentation was a palpable lump in the breast, with no other symptoms during the 10 years of follow‐up.

The definitive diagnosis of AME and myoepithelial carcinoma is based on histological and immunohistochemical findings.^2^ AME is a tumor consisting of epithelial and myoepithelial cells and is characterized by bicellular proliferation.^4^ Myoepithelioma is a tumor that consists of myoepithelial cells but lacks epithelial cells.^6^ The presence of an infiltrating border, necrosis, marked cytological atypia, and increased mitotic rate (>10 mitotic figures/10 HPF) are indicators of malignancy.^7^, ^8^ Myoepithelial carcinomas are diagnosed based on the proliferation of myoepithelial cells and other malignant indicators. Immunohistochemistry reveals that while these tumors are positive for CK5, CK14, CK17, p63, SMA, S‐100, and CD10, they are negative for CK18, CK19, and desmin.^9^ The differential diagnosis of myoepithelial carcinoma includes invasive ductal carcinoma, leiomyosarcoma, metaplastic carcinoma, spindle cell sarcoma, fibromatosis, and myofibroblastoma. While invasive ductal carcinomas are negative for p63, leiomyosarcomas typically show blunt‐ended nuclei with abundant cytoplasm and positivity for desmin. The metaplastic carcinomas show areas of metaplastic differentiation, such as cartilage, whereas the spindle cell sarcomas lack the epithelial component. Fibromatosis shows infiltrative fibroblastic and myofibroblastic proliferation.^9^ Myofibroblastomas, in contrast, are cellular, collagenized, epithelioid, palisaded, lipomatous, and hemangiopericytoma‐like, with infiltrant features. They are also positive for vimentin, CD34, and desmin.^10^

Adenomyoepitheliomas can undergo malignant transformation in either the myoepithelial or the epithelial component, or both and give rise to a myoepithelial carcinoma.^11^ In the present case, the pathogenesis of the tumor was described as involving “myoepithelial cells of AME” that underwent malignant transformation, based on ultrasonographic findings showing transformation (BI‐RADS category 2‐5), and increase in size, along with pathological findings indicative of the two components without separation.

In this case, the excision was delayed because of a misdiagnosis of the core needle biopsy and the patient's refusal to have an excisional biopsy. We misdiagnosed the core needle biopsy as a benign epithelial neoplasm. In previous reports, a sampling of the nonrepresentative areas or the presence of a myxoid background makes the diagnosis of a myoepithelial carcinoma less straight forward though it should be considered.^1^ Since we were unable to get specimens of the myoepithelial carcinoma, there was a discrepancy between the ultrasonographic findings of malignancy and the cytological findings.

The treatment of myoepithelial carcinoma is not yet established. There are 31 case reports and 4 case series reports published in English up till 2019, which include a total of 63 cases of myoepithelial carcinoma. The initial treatment for a small size tumor under 3 cm is usually lumpectomy or wide excision,^6^, ^12^, ^13^, ^14^, ^15^, ^16^ while that for a large size tumor over 3 cm is mastectomy with axillary lymph node clearance.^1^, ^2^, ^17^ On the other hand, the inoperative cases are selected for chemoradiotherapy.^18^, ^19^ While most cases of myoepithelial carcinoma have a good prognosis, a few of them show local recurrence or distant metastasis.^1^, ^2^ Adjuvant radiotherapy is often administered in order to minimize local recurrence.^19^ In this case, because of the small size of the tumor, its well‐defined border, and slow rate of growth, we chose breast conservation surgery. Additionally, sentinel node biopsy and adjuvant radiotherapy were performed to prevent lymph node metastasis and local recurrence. Careful follow‐up is required in the future.

## 4 CONCLUSIONS

We have presented a case of AME with myoepithelial carcinoma with follow‐up over 10 years. To the best of our knowledge, there are no other published reports that present the process of malignant transformation through ultrasonographic findings over a long period of time. This patient was treated using a combination of conservative surgery and radiation therapy. The definitive diagnosis of AME and myoepithelial carcinoma was based on histological and immunohistochemical findings. Early excisional biopsy and careful follow‐up are critical in such cases.

## CONFLICT OF INTEREST

None declared.

## AUTHOR CONTRIBUTIONS

RI: performed the operation, managed the perioperative course, and wrote the manuscript. DO: performed the operation, managed the perioperative course, and wrote the manuscript. SA: made the pathological diagnosis and wrote the manuscript. MM: made the pathological diagnosis and wrote the manuscript. AF: managed the perioperative course and approved the final manuscript.

## CONSENT FOR PUBLICATION

This patient consented to the reporting of this case in a scientific publication.
